# Targeting myeloperoxidase limits myeloid cell immunosuppression enhancing immune checkpoint therapy for pancreatic cancer

**DOI:** 10.1007/s00262-024-03647-z

**Published:** 2024-02-17

**Authors:** Angisha Basnet, Kaitlyn M. Landreth, Remi Nohoesu, Stell P. Santiago, Werner J. Geldenhuys, Brian A. Boone, Tracy W. Liu

**Affiliations:** 1 Department of Microbiology, Immunology, and Cell Biology, School of Medicine, West Virginia University, 64 Medical Center Drive, Morgantown, WV 26506 USA; 2 WVU Cancer Institute, West Virginia University, Morgantown, WV 26506 USA; 3 Department of Pathology, Anatomy and Laboratory Medicine, West Virginia University, Morgantown, WV 26506 USA; 4 Department of Pharmaceutical Sciences, West Virginia University, Morgantown, WV 26506 USA; 5 Division of Surgical Oncology, Department of Surgery, West Virginia University, Morgantown, WV 26506 USA

**Keywords:** Pancreatic cancer, Myeloid cells, Myeloperoxidase, Immune checkpoint therapy

## Abstract

Pancreatic ductal adenocarcinoma is a devastating disease characterized by an extreme resistance to current therapies, including immune checkpoint therapy. The limited success of immunotherapies can be attributed to a highly immunosuppressive pancreatic cancer microenvironment characterized by an extensive infiltration of immune suppressing myeloid cells. While there are several pathways through which myeloid cells contribute to immunosuppression, one important mechanism is the increased production of reactive oxygen species. Here, we evaluated the contribution of myeloperoxidase, a myeloid-lineage restricted enzyme and primary source of reactive oxygen species, to regulate immune checkpoint therapy response in preclinical pancreatic cancer models. We compared treatment outcome, immune composition and characterized myeloid cells using wild-type, myeloperoxidase-deficient, and myeloperoxidase inhibitor treated wild-type mice using established subcutaneous pancreatic cancer models. Loss of host myeloperoxidase and pharmacological inhibition of myeloperoxidase in combination with immune checkpoint therapy significantly delayed tumor growth. The tumor microenvironment and systemic immune landscape demonstrated significant decreases in myeloid cells, exhausted T cells and T regulatory cell subsets when myeloperoxidase was deficient. Loss of myeloperoxidase in isolated myeloid cell subsets from tumor-bearing mice resulted in decreased reactive oxygen species production and T cell suppression. These data suggest that myeloperoxidase contributes to an immunosuppressive microenvironment and immune checkpoint therapy resistance where myeloperoxidase inhibitors have the potential to enhance immunotherapy response. Repurposing myeloperoxidase specific inhibitors may provide a promising therapeutic strategy to expand therapeutic options for pancreatic cancer patients to include immunotherapies.

**Supplementary Information:**

The online version contains supplementary material available at 10.1007/s00262-024-03647-z.

## Introduction:

Pancreatic ductal adenocarcinoma (PDAC) is one of the most lethal cancer diagnoses, with less than 12% of patients surviving 5 years after detection [1]. Normally diagnosed at late stages, PDAC is an aggressive and devastating disease characterized by rapid progression and profound resistance to current therapies, including immune checkpoint therapy (ICT) [2, 3]. Currently, ICT is only available to the 1% of patients with high microsatellite instability [4]. The limited success of immunotherapies can be attributed to the highly immunosuppressive PDAC microenvironment characterized by an extensive infiltration of myeloid cells [5–10]. The presence of these myeloid cells has been strongly associated with poor prognosis and therapeutic outcome [10–12]. While there are several mechanisms that contribute to myeloid cell immunosuppression, one important process is the increased production of reactive oxygen species (ROS) [13–16]. Myeloperoxidase (MPO) is a myeloid-lineage restricted respiratory burst enzyme that is a major source of ROS [16–20]. MPO is central to innate immune cell microbial defenses by catalyzing the formation of hypochlorous acid during the phagocytic pathway in activated neutrophils [21]. We and others have demonstrated an increase in MPO in myeloid cells in the presence of cancer [16–18, 22]. MPO has been implicated in the inhibition of T cells, natural killer (NK) cells, and dendritic cells [18–20, 23]. Additionally, MPO has been shown to be involved in the recruitment and activation of additional myeloid cells [24, 25], further exacerbating their immunosuppressive effects. In this study, given the high infiltration of myeloid cells in the PDAC microenvironment, we evaluated the contribution of MPO in mediating ICT response in preclinical PDAC models. Our studies using a subcutaneous PDAC model with genetic knockouts and clinically translatable MPO inhibitors demonstrate that MPO regulates immunotherapy response and myeloid cell immunosuppression. Additionally, our data demonstrate an increase in MPO expression in pancreatic cancer compared to normal pancreas tissues. Our findings establish MPO as a promising therapeutic target with the potential to expand PDAC treatment options to include ICT.

## Results

### Host MPO deficiency enhances ICT response and immune composition.

**Fig. 1:**
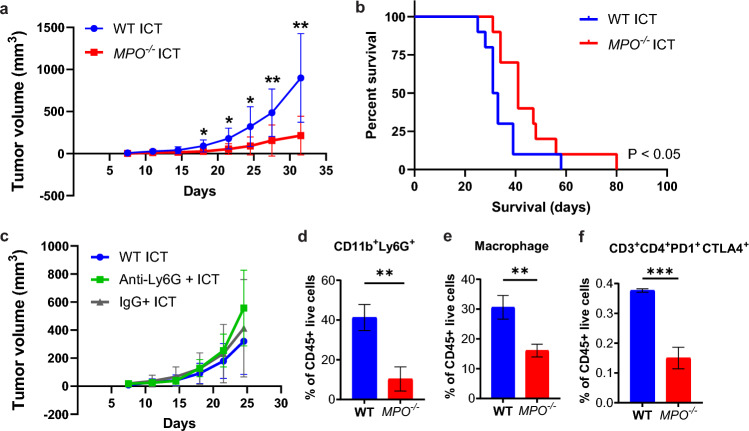
Tumor growth and immune composition of subcutaneous PDAC-bearing WT and *MPO*^*−/−*^ mice. **a** Tumor volume measurements and **b** survival curves for KPCY6419 tumor-bearing 8-week-old WT and *MPO*^*−/−*^ mice treated with ICT (n = 10 mice/group). **c** Tumor volume measurements of KPCY6419 tumor-bearing WT mice treated with anti-Ly6G and ICT (*n* = 5 mice/group). Flow cytometry of endpoint KPCY6419 **c**–**e** tumors from WT and *MPO*^*−/−*^ mice (*n* = 6 mice/group). Data shown as mean ± SD; unpaired Student’s t test, *p < 0.05, **p < 0.01, ***p < 0.001

We evaluated how MPO activity impacted the response of established PDAC tumors to ICT. Using a syngeneic immunocompetent subcutaneous tumor model, ICT efficacy was compared in wild-type C57BL/6 (WT) and age-matched syngeneic MPO-deficient (*MPO*^*−/−*^) animals using the KPCY6419 murine PDAC cell line [26]. Enhanced ICT response was observed, characterized by a significant delay in tumor growth and increased survival time, in ICT treated *MPO*^*−/−*^ mice compared to WT mice (Fig. 1a, b, S1a). No differences in KPCY6419 tumor growth or survival were observed between untreated PDAC-bearing WT and *MPO*^*−/−*^ mice (Fig. S1b, c). Additionally, no sex-based differences in tumor growth or survival between male and female WT and *MPO*^*−/−*^ mice treated with and without ICT were observed (Fig. S1d–g). Since MPO is the most abundant protein product in azurophilic granules of granulocytes [27], we evaluated how ICT response was affected by depletion of Ly6G^+^ myeloid cells using an anti-Ly6G antibody which primarily depletes granulocytes [28]. No difference in growth was observed in KPCY6419 tumors when treated with anti-Ly6G antibody and ICT or anti-Ly6G antibody alone (Fig. 1c and S1h). This data suggests that MPO itself was the predominant mediator of ICT response, rather than granulocytes.

### MPO activity affects immune composition.

The tumor-immune composition of KPYC6419 subcutaneous tumors from WT and *MPO*^*−/−*^ was evaluated using flow cytometry at tumor endpoint. Host MPO deficiency significantly reduced both CD11b^+^Ly6G^+^ myeloid cells, macrophages (CD11b^+^F4/80^+^) and exhausted CD4^+^PD1^+^CTLA4^+^ T cells within the tumor microenvironment (Fig. 1d–f). No difference was observed in other immune cell subsets, CD11b^+^ cells, CD8^+^ T cells, CD4^+^ T cells, T regulatory (Treg) cells, monocytes, natural killer (NK) cells, B cells, or dendritic cells (Fig. S2). Histology of endpoint tumors demonstrated a significant decrease in CD8^+^ T cells when MPO was deficient, but similar to flow cytometry, no changes in CD11b^+^ myeloid cells (Fig. S3). The systemic immune composition was also evaluated using the spleen of tumor-bearing WT and *MPO*^*−/−*^ mice. A statistically significant decrease in CD3^+^ T cells was observed when MPO was deficient (Fig. S4), specifically CD4^+^ T cells, PD1^+^CTLA4^+^CD4^+^, and PD1^+^CD8^+^ T cells as well as Treg cells. No difference in other immune cell subsets were observed in the spleen (Fig. S4).

**Fig. 2:**
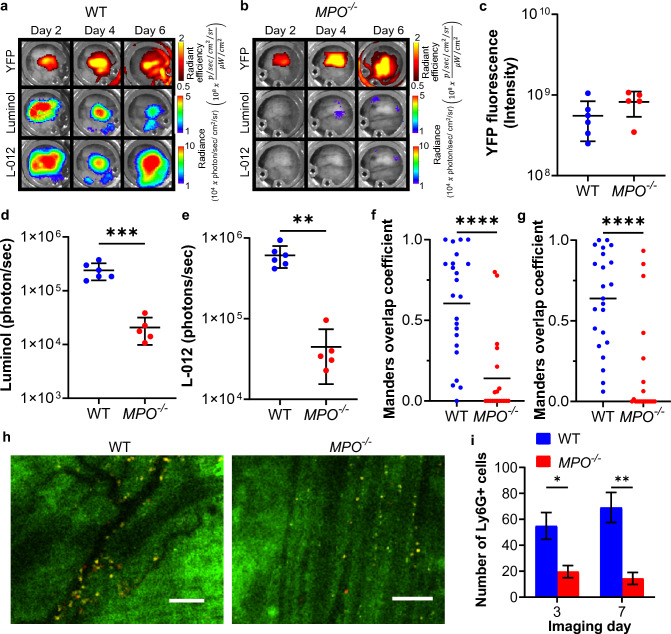
Intravital imaging of spatial and transient changes within the tumor microenvironment. Representative macroscopic intravital images of the same skin window chamber in KPCY6419 PDAC-bearing **a** WT and **b** *MPO*^*−/−*^ mice (*n* = 6 WT, *n* = 5 *MPO*^*−/−*^). Time average of mean of **c** YFP tumor fluorescence, **d** luminol and **e** L-012 photon flux. Pixel intensity spatial colocalization quantification (Manders’ overlap coefficients) of KPCY6419 tumor YFP fluorescence and proximate **f** luminol and **g** L-012 bioluminescence of MPO activity and ROS. **h** Representative intravital confocal image of KPCY6419 tumor (YFP, green), Ly6G^+^ myeloid cells (Ly6G^+^, yellow) and CD8^+^ T cells (CD8a^+^, red); scale bars, 100 μm. **i** Corresponding quantification of number of Ly6G^+^ cells within the tumor (*n* = 5 WT mice, *n* = 4 *MPO*^*−/−*^ mice; *n* = 3 images per mouse per time point). Data shown as mean ± SD; two-way ANOVA followed by Bonferroni's multiple comparisons test, *p < 0.05, **p < 0.01

Using intravital imaging with skinfold window chamber murine models [29, 30], we evaluated changes in the MPO activity and ROS levels within the tumor microenvironment during KPCY6419 tumor growth in real-time (Fig. 2a, b). KPCY6419 cells stably expressed a constitutively active yellow fluorescent protein (YFP) allowing for imaging of tumor mass and location. Similar to the subcutaneous studies, no difference in KPCY6419 tumor growth quantified using YFP fluorescence was observed between WT and *MPO*^*−/−*^ skin window chamber bearing mice (Fig. 2c, Fig. S5a). Luminol and L-012 bioluminescence imaging were used to assess the level of enzymatic MPO activity and ROS levels, respectively, from infiltrating myeloid cells in real time in vivo (Fig. 2a, b) [29–31]. As expected, increased MPO activity and ROS levels were quantified during PDAC progression in WT compared to *MPO*^*−/−*^ window chamber bearing mice (Fig. 2d, e, Fig. S5 b, c). Pixel intensity spatial colocalization of KPCY6419 tumor YFP fluorescence and proximate luminol or L-012 bioluminescence was evaluated. WT mice demonstrated Manders’ overlap coefficients that approached 1, suggesting that MPO-active and ROS producing myeloid cells infiltrated the tumor microenvironment during PDAC progression (Fig. 2f, g). In contrast, Manders’ overlap coefficients for YFP and luminol or L-012 approached 0 when MPO was deficient. Using intravital microscopy, a significant decrease in Ly6G^+^ myeloid cells was observed when MPO was deficient (Fig. 2h, i). No statistical difference was observed in the early infiltration of CD8^+^ T cells (Fig. S5d). These intravital imaging studies mirrored the subcutaneous tumor endpoint flow cytometry data.

### MPO activity affects myeloid cell functions

**Fig. 3:**
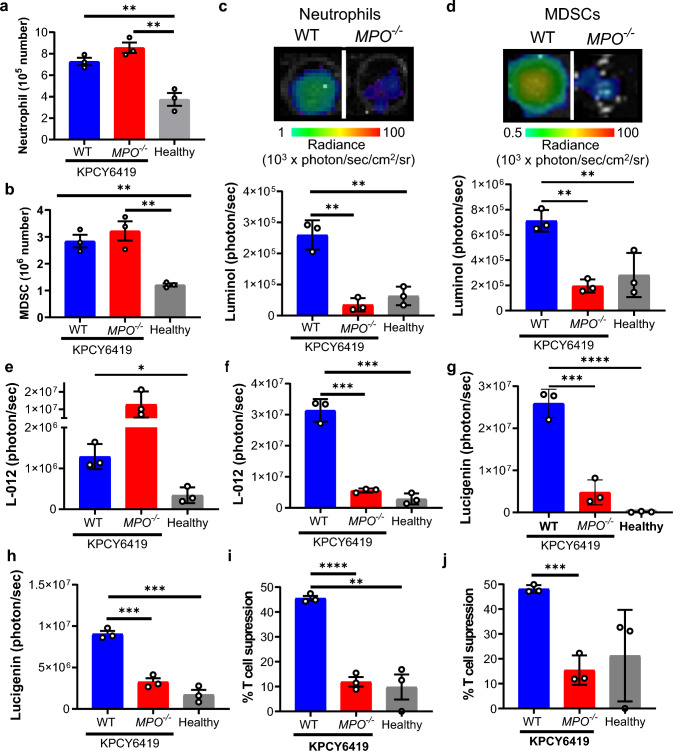
Isolated neutrophils and MDSC characterization. Isolated number of **a** neutrophils and **b** MDSCs from the spleens of tumor-bearing WT and MPO^−/−^ mice and healthy (tumor-free) WT mice. Representative bioluminescence image and corresponding quantification of luminol in **c** neutrophils and **d** MDSCs. Quantification of L-012 bioluminescence in **e** neutrophils and **f** MDSCs and lucigenin bioluminescence in **g** neutrophils and **h** MDSCs. CD8^+^ T cell suppression by **i** neutrophils and **j** MDSCs at 72 h post-co-culture (data normalized to CD3, CD28 activated CD8^+^ T cells alone at 72 h). Data shown as mean ± SD; *n* = 3 mice/group. One-way ANOVA followed by Tukey’s multiple comparison test, *p < 0.05, **p < 0.01, ***p < 0.001, ****p < 0.0001

While MPO is primarily expressed by neutrophils, myeloid-derived suppressor cells (MDSCs), which are pathologically activated immature neutrophils, have also been shown to express MPO [17, 18]. Both neutrophils and MDSCs also express CD11b^+^ and Ly6G^+^ surface markers. Therefore, we isolated both neutrophils and MDSCs from the spleen of tumor-bearing WT and *MPO*^*−/−*^ mice. The presence of KPCY6419 tumors increased total number of isolated neutrophils and MDSC in the spleen of both WT and *MPO*^*−/−*^ mice compared to WT healthy (tumor-free) mice (Fig. 3a, b). The purity of the isolated neutrophils and MDSCs was confirmed by flow cytometry (Fig. S6a, b). MPO activity and ROS levels were quantified using the bioluminescence reporters luminol, L-012 and lucigenin. Luminol measures MPO activity, while L-012 quantifies general ROS levels including MPO [29–31]. As expected, neutrophils and MDSCs isolated from tumor-bearing WT mice had a statistically significant increase in MPO activity compared to tumor-bearing *MPO*^*−/−*^ or healthy (tumor-free) mice quantified using luminol bioluminescence (Fig. 3c, d). Interestingly, no difference in ROS levels using L-012 was observed between isolated neutrophils from tumor-bearing WT and *MPO*^*−/−*^ mice but a significant increase in neutrophil ROS was observed from tumor-bearing WT mice compared to healthy mouse controls (Fig. 3e). However, isolated MDSCs from tumor-bearing WT mice had a significant increase in ROS levels by L-012 bioluminescence compared to tumor-bearing *MPO*^*−/−*^ and healthy WT mice, mirroring the luminol signal (Fig. 3f). Using the bioluminescence reporter lucigenin [32], production of the ROS superoxide anion was measured; both isolated neutrophils and MDSCs demonstrated significant increase in superoxide anion levels in WT tumor-bearing mice compared to tumor-bearing *MPO*^*−/−*^ and healthy WT mice (Fig. 3g, h). Both neutrophils and MDSCs isolated from tumor-bearing WT mice had statistically significant increase in suppression of CD8^+^ T cell proliferation compared to neutrophils and MDSCs isolated from tumor-bearing *MPO*^*−/−*^ and healthy WT mice (Fig. 3i, j). A significant increase in extracellular trap formation, as measured by release of cell-free DNA in cultured supernatant, was only observed from MDSCs isolated from tumor-bearing WT mice compared to tumor-bearing *MPO*^*−/−*^ and healthy mice, while neutrophils did not demonstrate any different in extracellular trap formation (Fig. S6c, d). These data suggest that MPO contributes to neutrophil and MDSC ROS production, T cell suppression, and extracellular trap formation.

### Pharmacological Inhibition of MPO enhances ICT response.

**Fig. 4:**
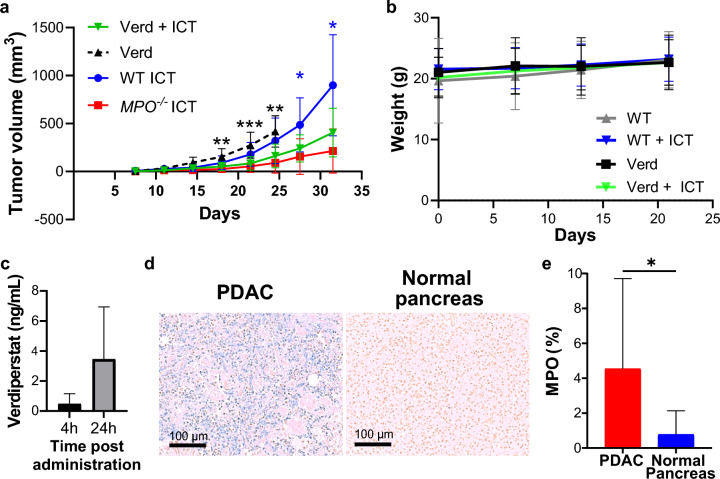
PDAC-bearing mice treated with combination ICT and MPO inhibitor verdiperstat. **a** Tumor volume and **b** body weight measurements for KPCY6419 tumor-bearing 8-week-old WT treated with verdiperstat (verd) with or without ICT (*n* = 10 mice/group). Data shown as mean ± SD, one-way ANOVA followed by Dunnett’s multiple comparison test, *p < 0.05, **p < 0.01, ***p < 0.0001 (blue * indicates significant differences between WT ICT and verd + ICT, black * indicates significant differences between verd and verd + ICT). **c** Concentration of verdiperstat measured by mass spectroscopy in KPCY6419 tumors at 4 h and 24 h post-single IP injection (*n* = 3 mice/group). MPO expression in pathological human samples. **d** Representative histology of MPO staining in human PDAC and normal pancreas samples and **e** corresponding quantification of percent of cells with MPO expression quantification; human tissue microarrays (US Biomax PA483e and PA961f; *n* = 109 PDAC, *n* = 13 normal pancreas includes normal, adjacent, or cancer adjacent skin tissues). Data shown as mean ± SD, unpaired Student’s *t* test, *p < 0.05

To simulate clinical application, the efficacy of combination ICT and MPO inhibitors in WT animals bearing KPCY6419 tumors was evaluated. We used two different MPO inhibitors: verdiperstat and AZD5904, in which treatment began 1 day prior to ICT treatment. Combination treatment with verdiperstat and ICT significantly delayed tumor growth compared to ICT alone to levels equivalent to host MPO gene deficiency (Fig. 4a and S7a). Mice tolerated verdiperstat well without apparent side effects as body weight was not affected (Fig. 4b). A concentration of 0.5 ± 0.7 ng/mL and 3.5 ± 3.4 ng/mL was measured in subcutaneous KPCY6419 tumors at 4 h and 24 h post-intraperitoneal injection, respectively (Fig. 4c). AZD5904 treatment significantly delayed tumor growth when combined with ICT compared to AZD5904 alone but did not significantly delay tumor growth compared to ICT alone (Fig. S7b, c). Mice tolerated AZD5904 treatment as well without apparent side effects or change in body weight (Fig. S7d). No differences in tumor growth were observed between male and female mice when treated with MPO inhibitors (Fig. S7e, f). Inhibition of MPO activity by verdiperstat and AZD5904 was confirmed using phorbol myristate acetate (PMA)-stimulated bone marrow isolated immune cells. Significant decreases in luminol bioluminescence signal were observed when PMA-stimulated bone marrow isolated immune cells were incubated with verdiperstat or AZD5904 (Fig. S8).

### MPO expression in pathological samples

MPO staining of two human pancreas tissue arrays demonstrated varying levels of MPO protein (Fig. 4d and S9). In contrast, normal pancreas tissue (including normal pancreas tissue and cancer adjacent pancreas tissue) demonstrated minimal MPO staining. MPO expression was significantly increased in pancreatic cancerous tissues compared to normal samples (Fig. 4e). Using the TCGA data set for pancreatic adenocarcinoma with the TIMER2.0 XCell algorithm [33], we observe a significantly positive correlation of MPO gene expression with immunosuppressive subsets of immune cells including T regulatory cells, Th2 CD4^+^ T cells, M2 macrophages and cancer associated fibroblasts (Fig. S10 a–e), and a negative correlation with anti-tumor Th1 CD4^+^ T cells (Fig. S10f). As expected, MPO expression significantly positive correlated with myeloid cells (Fig. S10g). We additionally found that MPO expression had no correlation to CD8^+^ T cells (Fig. S10h), but observed a positive correlation to M1 macrophages (Fig. S10i). These data demonstrated the presence and increase in MPO in clinically relevant human pancreatic cancer tissues compared to normal pancreas tissues and highlight a translational potential for MPO targeting in PDAC.

## Discussion

It is evident that there is an urgent need for advancements in the treatment of PDAC, a disease with poor prognosis and few therapeutic options. The PDAC microenvironment is characterized by an increased infiltration of myeloid cells where the ability to modulate these cells has the potential to enhance therapeutic responses. In this study, we report that genetic deficiency and pharmacological inhibition of MPO enhanced ICT efficacy in a subcutaneous model of PDAC. Our data demonstrated significant delay in tumor growth using an MPO specific inhibitor, verdiperstat, combined with ICT compared to ICT or inhibitor alone. When ICT was combined with verdiperstat, an equivalent response similar to host MPO deficiency was observed. While the use of two clinically relevant MPO selective inhibitors, verdiperstat and AZD5904, was evaluated, combination AZD5904 with ICT did not demonstrate similar enhanced response as with verdiperstat. However, only a single treatment dose for combination MPO inhibition with ICT was evaluated. Future optimization of the dosing regimen of MPO inhibition, particularly using AZD5904, may be required in order to observe enhanced ICT outcome. The clinical use of these MPO inhibitors is feasible and evaluated clinically for Parkinson’s disease, multiple system atrophy and amyotrophic lateral sclerosis [34–37]. These data provide a preclinical foundation where MPO selective inhibitors may have therapeutic repurposing potential expanding the therapeutic options for PDAC to include immunotherapy when used in combination with MPO inhibition. The overexpression of MPO documented in human PDAC samples further highlights this opportunity. These findings are particularly significant for PDAC in which new therapeutic approaches are needed due to its dismal overall survival.

In our model, depletion of Ly6G^+^ cells did not improve ICT efficacy, suggesting that MPO specifically is the prominent mediator of ICT response rather than a subset of myeloid cells. Only when MPO was deficient or inhibited did we see enhanced ICT response, indicating that MPO itself contributes to ICT resistance. Furthermore, this data suggests that MPO is expressed by other myeloid cell subsets beyond Ly6G^+^ cells. Using flow cytometry, the tumor and systemic immune composition was evaluated. MPO deficiency significantly reduced CD11b^+^Ly6G^+^ myeloid cells, macrophages and exhausted CD4^+^PD1^+^CTLA4^+^ T cells within the tumor microenvironment. No difference in CD11b^+^Ly6G^+^ myeloid cells or macrophages was observed in the spleen. These data suggest that MPO may contribute to recruitment and/or infiltration of CD11b^+^Ly6G^+^ myeloid cells and macrophages within the tumor microenvironment, but not systemically. However, the mechanism by which MPO activity recruits these myeloid cells into the tumor microenvironment requires further studies. Similar to the tumor microenvironment, a significant decrease in CD4^+^ PD1^+^ T cells in the spleen was observed as well as exhausted CD8^+^ T cells and T regulatory cells when MPO was deficient. These data suggest that MPO activity may mediate T cell exhaustion and T regulatory cells, T cell subsets that influence an immunosuppressive microenvironment and ICT response. However, the mechanism by which MPO enhances T cell exhaustion and/or recruits T regulatory cells requires additional evaluation. Differences in T cell subsets were observed between the tumor and spleen. This may be a result of differences in T cell homing between WT and *MPO*^*−/−*^ mice. Further studies are underway to evaluate whether MPO contributes to T cell recruitment in a tissue site specific manner. Intravital imaging studies further confirmed the decrease of Ly6G^+^ myeloid cells with in the tumor microenvironment. Using luminol and L-012 bioluminescence imaging, intravital imaging in *MPO*^*−/−*^ mice had decreased MPO activity (as expected) and reduced ROS levels within the tumor microenvironment further suggesting that MPO deficiency resulted in a less immunosuppressive microenvironment. The quantification and colocalization of MPO activity and ROS levels using luminol and L-012 primarily reflects the contribution of infiltrating immune cells rather than the tumor cells themselves. This is evident from *MPO*^*−/−*^ mice, which underwent identical tumor cell injections, revealing minimal levels of detectable ROS that did not colocalize with the tumor. Despite similar findings in flow cytometry of endpoint tumors and real-time intravital imaging, which showed no variance in CD8^+^ T cells between WT and *MPO*^*−/−*^ mice, histological examination revealed a reduction in CD8^+^ T cells when MPO was deficient. This disparity may arise from the limited scope of our histological analysis which evaluated a single tumor slice. This sampling might not accurately mirror the overall T cell infiltration across the entire tumor due to the inherent heterogeneity within the tumor microenvironment. While the influence of MPO on T cells and the specific mechanism underlying MPO-dependent cell recruitment requires further studies, our data demonstrate a significant decrease in CD11b^+^Ly6G^+^ myeloid cells within the tumor microenvironment when MPO is deficient using multiple methods.

To delineate the possible mechanisms of MPO-mediated ICT enhancement, we isolated CD11b^+^Ly6G^+^ subsets from the spleen of tumor-bearing and healthy mice. Since both neutrophils and MDSCs express these surface markers, both subsets were isolated. The total number of spleen isolated neutrophils (CD11b^+^Ly6G^+^) and MDSCs (CD11b^+^Gr1^+^) was increased in the presence of PDAC. Generally, MPO activity and ROS levels were increased in neutrophils and MDSCs isolated from WT mice compared to *MPO*^*−/−*^ mice. Our data demonstrate that loss of MPO results in a substantial decrease in the ability of neutrophils and MDSCs to suppress CD8^+^ T cell proliferation. This finding strongly reinforces the observation that immune suppressing myeloid cells affect T cells through MPO [19, 20]. Whether MPO activity in neutrophils or MDSCs suppress other immune cells subsets requires further evaluation. It should be noted that our MDSC subset is a combination of Ly6G^+^ and Ly6C^+^ cells and additional studies are underway to evaluate if MPO similarly regulates the function of Ly6G^+^ and Ly6C^+^ MDSC subsets. Interestingly, loss of MPO resulted in a reduction of extracellular trap formation only in MDSCs and not neutrophils. However, these studies were evaluated in basal cells and the ability of neutrophils and MDSCs to form extracellular traps through stimulation was not evaluated. Taken together, this data suggests that limiting MPO results in a reduction in the degree of immunosuppression within the microenvironment, which may be the underlying mechanism driving the observed enhanced ICT response when MPO is deficient or inhibited. While improved immunotherapy outcomes have been observed by decreasing the infiltration of myeloid cells into the PDAC tumor microenvironment [38–40], our studies demonstrate that by targeting MPO, we both enhance ICT efficacy and also limit immunosuppression in myeloid cells.

Our flow cytometry and intravital imaging studies demonstrated low levels of CD8^+^ T within the tumor microenvironment. Our data support the observation that KPCY6419 tumors have minimal infiltrating T cells. [26] Interestingly, flow cytometry of endpoint tumors and real-time intravital imaging of early tumor time points demonstrated no differences in CD8^+^ T cell infiltration between WT and *MPO*^*−/−*^ mice. This low number of T cells within the tumor microenvironment could account for the absence of discernible differences in KPCY6419 tumor growth in untreated WT and *MPO*^*−/−*^ mice. Although MPO deficiency results in limiting and loss of immunosuppressive myeloid cells causing in an overall reduction in immunosuppression within the tumor microenvironment, the low infiltration of T cells may not be sufficient to elicit an anti-tumor immune response in *MPO*^*−/−*^ mice. Only with ICT treatment, when T cells are activated, are differences in tumor growth detected, highlighting the contribution of MPO in shaping an immunosuppressive microenvironment. Our data also suggest that MPO does not contribute to the infiltration of CD8^+^ T cells within the tumor microenvironment. However, additional studies are required to evaluate how MPO contributes to PDAC progression using high T cell infiltrated KPC cell lines [26].

While our data have provided valuable insights, it is essential to acknowledge that there are several limitations in our studies. We recognize that our ex vivo studies only characterized spleen isolated myeloid cells; unfortunately, it proved challenging to isolate high purity myeloid cells from PDAC tumors (Fig. S11). Additionally, our studies used a subcutaneous tumor model which does not fully recapitulate the complex PDAC microenvironment during primary tumor growth or metastatic disease. While we observed various immune infiltrates in our subcutaneous tumors, the immune cells found in subcutaneous tumors may not be the best representation of the immune composition of orthotopic PDAC tumors or metastases. Ongoing studies are under evaluation to understand the contribution of MPO in orthotopic and spontaneous PDAC preclinical models. While our data demonstrate that MPO suppressed CD8^+^ T cell proliferation, the specific mechanism by which MPO mediates this suppression requires further studies. Given the decrease in exhausted T cells when MPO was deficient, MPO could suppress T cells through upregulation of exhaustion markers. Additionally, our previous work demonstrated that MPO inhibited tumor cell NF-κB signaling [31] and we anticipate that MPO could similarly inhibit NF-κB signaling in T cells, decreasing T cell activation. Furthermore, the major product of MPO is hypochlorous acid (HOCl), a potent oxidating agent. MPO produced HOCl may limit T cells by causing T cell apoptosis. Studies are underway to understand the specific mechanism by which MPO suppresses T cells. We further recognize that the *MPO*^*−/−*^ mice are global genetic knockouts. However, MPO within the host is restricted to myeloid-lineage cells and we expect, as previously reported, that other host immune cells will function normally [41]. While our data also demonstrated a decrease in macrophages when MPO was deficient, MPO has been reported to be found in much smaller quantities in macrophages [21]. In the context of atherosclerosis, an increase in macrophages with high levels of MPO was observed in eroded or ruptured plaques [42]. Additional studies are required to evaluate whether MPO in macrophages is altered in PDAC and how MPO activity alters macrophage function. It is also possible the difference in macrophages observed in MPO-deficient mice is a consequence of a decrease in CD11b^+^Ly6G^+^ myeloid cells.

Overall, our findings support combination MPO inhibition and ICT as a new treatment approach for delaying PDAC tumor growth. Histological analysis of MPO staining in human tissue arrays demonstrated increased MPO expression in PDAC compared with normal pancreas tissues suggesting that MPO has potential to be a clinically relevant target. Since MPO is a myeloid-lineage restricted enzyme, targeting MPO has built-in specificity for myeloid cells broadly. The safety, tolerability and pharmacokinetics of MPO inhibitors in humans have been assessed with minimal reported toxicities [34]. Additionally, individuals with clinical manifestations of MPO deficiency typically exhibit mild or asymptomatic conditions, and they generally do not experience an elevated frequency of infections [43, 44]. Taken together, the use of MPO inhibitors should exhibit minimal on-target toxicities. In summary, our work using a subcutaneous PDAC model with genetic knockouts and clinically translatable MPO inhibitors establish MPO as a promising therapeutic target with the potential to expand PDAC treatment options to include ICT.

## Materials and methods

### Reagents

Luminol (sodium salt), and lucigenin (*N*,*N*′-Dimethyl-9,9′-biacridinium dinitrate) were purchased from Sigma-Aldrich (Sigma-Aldrich, MO, USA). L-012 sodium salt was purchased from Wako Chemicals USA (Wako Chemicals USA, Inc., Richmond, VA, USA). Verdiperstat, AZD5904 and hydroxypropyl-*β*-cyclodextrin (HP-*β*-CD) were purchased from MedChemExpress (MedChemExpress LLC, NJ, USA). Anti-CTLA-4 (9D9), anti-αPD-1 (RMP1-14), anti-Ly6G (1A8) and polyclonal rat IgG control were obtained from Bio X cell (Bio X cell, NH, USA). Luminol sodium salt was dissolved in sterile phosphate-buffered saline (PBS) to a final concentration of 50 mg/mL or 100 mg/mL and stored at − 20ºC. L-012 powder was dissolved in sterile double distilled water (ddH_2_0) to a final concentration of 20 mM and stored at − 20ºC. Lucigenin was dissolved in sterile PBS to a final concentration of 1 mg/mL and made fresh for each imaging session. Verdiperstat and AZD5904 were dissolved in sterile DMSO to a final concentration of 10 mM and stored at − 20ºC; AZD5904 – 5% DMSO, 15% HP-β-CD w/v, 200µL intraperitoneal injection daily, Verdiperstat – 4% DMSO,15% HP-β-CD w/v, 300µL intraperitoneal injection daily.

### Cells

KPCY6419 cells were purchased from Kerafast (Kerafast, MA, USA). Cells were cultured in DMEM supplemented with 10% heat-inactivated fetal bovine serum (FBS) and 1% glutamine. Cell cultures were grown at 37ºC in a humidified 5% CO_2_ atmosphere. All cells lines tested negative for mycoplasma.

### In vivo subcutaneous tumor model

The Institutional Animal Care and Use Committee at West Virginia University approved all animal protocols (2109047227). 2 × 10^5^ KPYC6419 cells were injected subcutaneously on the right flank of male and female 8-week-old C57BL/6 (The Jackson Laboratory, ME, USA) or 8-week-old age-matched syngeneic C57BL/6 myeloperoxidase-deficient *MPO*^*−/−*^ (MPO^tm1Lus^, The Jackson Laboratory, ME, USA) animals. Prior to tumor cell injection, the fur was removed on the right flank. Tumors were measured by calipers ever 3–4 days once palpable. Following institutional animal guidelines, animals were euthanized once tumors reached 1.5 cm in diameter or were ulcerated greater than 0.5 cm. Animals were treated with ICT (200 µg Anti-CTLA-4 and 200 µg anti-PD-1 beginning on day 6 post-tumor inoculation every 3 days for 7 doses) [26, 45]. For the immune cell depletion studies, antibodies against Ly6G were injected into animals (400 µg in 100 µL PBS intraperitoneally) twice weekly for 3 weeks beginning on day 5 post-tumor inoculation (1 day before ICT treatment). Rat IgG isotype antibody was used as a control. For MPO inhibition studies, animals were injected intraperitoneally with 180 µmol/kg Verdiperstat or 180 µmol/kg AZD5904 daily[22] for 14 days beginning on day 5 post-tumor inoculation (1 day before ICT treatment). Survival curve statistical significance calculation was alpha-adjusted for multiple comparisons. Comparison of survival curves used the log rank (Mantel-Cox) analysis.

### Flow cytometry

At tumor endpoint, animals were euthanized by carbon dioxide asphyxiation. Single cell suspension of cells was harvested from the spleen and tumor. Tumors were dissociated using a mouse tumor dissociation kit (Miltenyi Biotec, CA, USA). Red blood cells are lysed using 1X red blood cell lysis buffer (Thermo Fisher Scientific, CA, USA). Remaining cells are resuspended in cell staining buffer (BioLegend, CA, USA) at a concentration of 2 × 10^5^–1 × 10^6^ cells per 100 µl. Fc receptors are blocked using 10 µg/ml ChromPure of mouse IgG (Jackson ImmunoResearch Inc., PA, USA) per 10^6^ cells in a 100 µl volume for 10 min on ice. Cells are washed with cell staining buffer and incubated with antibody mix (Table S1) for 15–20 min on ice in the dark. Cells are fixed using 200 µl Fixation buffer (Thermo Fisher Scientific, CA, USA) for 20 min at 4ºC in the dark. Cells are washed 2 times with 1X PermWash (BD Bioscience, CA, USA) and permeabilized using permeabilization solution (BD Bioscience, CA, USA). Intracellular antibodies (Table S1) were incubated for 20 min at room temperature in the dark. Stained cells were analyzed using the Cytek® Aurora (Cytek, MD, USA) within 2 weeks of staining. Data were analyzed using FCS express (De Novo Software, CA, USA). Figure S12 shows a representative flow cytometry gating scheme identifying the various immune cell subsets.

### Intravital imaging

Skin window chamber implantation, imaging and analysis were previously described using 8-week-old male and female WT and *MPO*^*−/−*^ animals [29–31]. Briefly, following skin window chamber implantation and KPCY6419 PDAC cell inoculation, macro-imaging occurred at day 2 post-implantation using the IVIS spectrum (PerkinElmer, Waltham, MA). Fluorescence imaging of tumors stably expressing YFP was performed before luminol (200 mg/kg of body weight) and L-012 bioluminescence imaging (25 mg/kg of body weight). Imaged was acquired 9 min post each bioluminescence substrate injection (acquisition time, 5 min; binning, 8; FOV, 6.6; f/stop, 1; filter, open). Quantifying the bioluminescence of window chamber animals occurred as follows. A background ROI was drawn at every imaging time point to measure the background bioluminescence. The bioluminescence within the window of individual mice was quantified by drawing a uniform background-subtracted ROI around the 1.2-cm glass coverslip at every imaging time point. Using a background-subtracted ROI to measure the bioluminescence of the reporters within the window chamber accounts for any fluctuations in noise at every imaging time point where signal output is the measured reporter’s photon flux above background. Colocalization analysis was performed on ImageJ using JACoP [46] plugin following reorientation and cropping using our previously described ImageJ macro [29].

### Intravital microscopy

Skin window chamber microscopy was done using a Nikon confocal AX microscopy (Nikon, NY, USA) at day 3 and day 7 post-skin window chamber implantation. 100 μL of antibody mixture per mouse: 0.15 µg Ly6G-PE (Miltenyi, CA, USA) and 0.15 µg CD8a-APC (Miltenyi, CA, USA) in PBS, was administered through retro-orbital injection. Microscopic imaging occurred 30 min post-antibody injection using 3 fields of view per mouse at 20X objective. KPCY6419 tumors were visualized within the skin window chamber using YFP. Images were analyzed using the Nikon Elements software (Nikon, NY, USA) using the automated measurement analysis macro. An individual threshold was applied to each imaging channel, and positive Ly6G-PE or CD8a-APC positive cells were quantified using the following analysis parameters; size: 5 µm to 10 µm, circularity: 0 to 1, smooth: 2X to 4X, fill holes: on and clean: 2X. The average of the 3 fields of view from each imaging time point for each animal was calculated.

### MDSC and neutrophil cell isolation

At tumor endpoint, animals were euthanized by carbon dioxide asphyxiation. Single cell suspension of cells was harvested from the spleen. MDSCs were isolated using an EasySep™ mouse MDSC isolation kit (STEMCELL Technologies Inc., MA, USA), and neutrophils were isolated using a mouse neutrophil isolation kit (Miltenyi Biotec, CA, USA); these cells were used for the MPO activity and ROS levels quantification, and T cell proliferation suppression. MDSCs were quantified by flow cytometry using CD11b-APC (Miltenyi Biotec, CA, USA) and Gr-1-PE (Miltenyi Biotec, CA, USA). Neutrophils were quantified by flow cytometry using CD11b-APC (Miltenyi Biotec, CA, USA) and anti-Ly6G-PE (Miltenyi Biotec, CA, USA).

### T cell proliferation assay

CD8^+^ T cells were isolated from the spleen of male healthy C57BL/6 mice using an EasySep™ mouse CD8^+^ T cell isolation kit (STEMCELL Technologies Inc., MA, USA). 1 × 10^6^ isolated T cells/mL were stained with 5 μM concentration of CellTrace CFSE (Thermo Fisher Scientific, CA, USA) and incubated at 37 °C for 20 min. Cells were incubated for 5 min with culture media to remove unbound dye and subsequently washed 2 times. CSFE stained T cells were incubated for 2–3 h at 37ºC in a humidified 5% CO_2_ atmosphere at a final concentration of 1 × 10^5^ isolated cells/ 100 μL. The following controls were incubated using a 1:1 bead-to-cell ratio of mouse T cell activation CD3/CD28 dynabeads (5 μL, Thermo Fisher Scientific, CA, USA) and immediately fixed using 0.4% paraformaldehyde overnight after CSFE staining of T cells: 2 × 10^5^ unstained (no CSFE) T cells, 2 × 10^5^ CSFE stained T cells (time zero, *t* = 0), 2 × 10^5^ MDSCs/neutrophils. Using a 1:1 ratio, 2 × 10^5^ T cells were cultured with 2 × 10^5^ MDSCs or neutrophils in 500 μL of RPMI Medium 1640 with 2 mM L-Glutamine, 10% FBS, and 100 U/ml penicillin/streptomycin and 5 μL dynabeads in 24-well plates for 72 h at 37ºC in a humidified 5% CO_2_ atmosphere. After 72 h, the 24-well plate was washed 2 times with culture media and the cell pellets were fixed using 0.4% paraformaldehyde overnight in the dark. T cell proliferation was quantified using the BD LSRFortessa™ Cell Analyzer (BD Biosciences, NJ, USA). Data were analyzed using FCS express (De Novo Software, CA, USA).

### MPO activity and ROS levels

MPO activity was assessed by luminol imaging of the isolated MDSCs and neutrophils; 1 × 10^5^ isolated cells were added to 100 mM luminol, 50 μM L-012 or 100 μM lucigenin in colorless DMEM media in a 96-well black walled plate. Cells were immediately imaged for 60 min using the Kino imaging system (Spectral Instruments Imaging, AZ, USA) at 37ºC under 5% CO_2_ flow. Typical acquisition parameters were as follows: acquisition time, 5 min; binning, 16; FOV, 13 cm; f/stop, 1.2; filter, open; total number of acquisitions 12. Bioluminescence photon flux (photons/sec) data were analyzed by ROI measurements with background subtraction in Aura (Spectral Instruments Imaging, AZ, USA); these raw data were imported into Excel (Microsoft Corp., WA, USA), averaged in each individual experiment if done in duplicate or triplicate wells. The area under the curve was calculated from images taken at 5–60 min. The area under the curve for each reporter was taken from *n* = 3 independent isolation experiments. MPO inhibition efficacy of verdiperstat and AZD5904 was measured using 100 mM luminol. 1 × 10^5^ bone marrow isolated immune cells from healthy wild-type mice were added to 100 mM luminol without or with 500 nM phorbol myristate acetate (PMA) to increase MPO activity, without or with 1 mM verdiperstat or AZD5904. Cells were immediately imaged for 30 min using the Kino imaging system (Spectral Instruments Imaging, AZ, USA) at 37ºC under 5% CO2 flow using the acquisition parameters described above. Luminol bioluminescence from the 10 min imaging time point was quantified for each group using *n* = 3 wells.

### Extracellular trap quantification

1 × 10^5^ isolated cells in 100 μL RPMI 1640 media were incubated for 1–2 h in a 96-well plate. After incubation, the cells were spun at 1000 rpm for 10 min. 50 μL of supernatant was removed and mixed with 50 μL of 1X TE buffer (Thermo Fisher Scientific, CA, USA) in a 96-well black walled clear bottom plate. 50 μL of 1:200 dilution of Pico Green in 1X TE buffer was added to each well, and fluorescence (Excitation: 480 nm and Emission: 520 nm) was quantified on a Synergy HTX multi-mode plate reader (BioTex, CA, US). A standard curve was made using the lambda DNA standard (Thermo Fisher Scientific, CA, USA) in 1X TE buffer (concentration range from 1 μg/ml to 25 pg/ml). The DNA concentration of sample was determined from the generated DNA standard curve.

### Tumor verdiperstat quantification

2 × 10^5^ KPYC6419 cells were injected subcutaneously on the right flank of 8-week-old C57BL/6 mice. When tumors reached approximately 5 mm in diameter, 180 µmol/kg verdiperstat was injected intraperitoneally. At 4 and 24 h post-injection, the tumor was excised and stored at – 80° C for further quantification. Verdiperstat was isolated from the tumor by addition of phosphate buffer saline pH 7.4 to the tumors followed by 10 s of sonication. Ethyl acetate was added to the samples, allowed to incubate using rotation for 1 h. The samples were centrifuged at 10,000 × *g* for 10 min and the ethyl acetate removed, evaporated and samples reconstituted in acetonitrile. Mass spectrometry using an ABSciex 5500 LC–MS/MS quantifies the verdiperstat of content within the tumor using a standard curve.

### MPO staining of tissue microarrays

Human PDAC and pancreas tissue arrays, PA483e and PA961f (US Biomax, MD, USA), were stained for MPO expression by the West Virginia University Pathology Research Laboratory core. MPO positive cell quantification was carried out by Dr. Stell Santiago. For MPO expression analysis, only fully stained cores for PDAC and normal pancreas tissues were compared; *n* = 109 PDAC samples and *n* = 13 normal pancreas samples.

### TCGA dataset

Using the TIMER2.0 XCell algorithm [33], the TCGA pancreatic adenocarcinoma (PAAD) data were used to evaluate the correlation of MPO expression with various immune cell infiltrates. The TIMER2.0 XCell algorithm generates the correlation graphs with correlation (rho) and *p* values.

### Statistical analyses

Graphs were made and statistical analyses were performed using GraphPad Prism (GraphPad Software, Inc., CA, USA). Data were expressed as mean ± SD. For analysis of three or more groups, analysis of variance (ANOVA) tests were performed followed by a Tukey’s or Bonferroni’s multiple comparisons test, or false discovery rate test. Standard comparison of survival curves used the log rank (Mantel–Cox) analysis. Analysis of differences between two normally distributed paired test groups was performed using a Student’s *t* test. *P* values were considered statistically significant if *p* < 0.05.

### Supplementary Information

Below is the link to the electronic supplementary material.

Supplementary file1 (DOCX 7396 KB)

## Data Availability

The datasets generated during and/or analyzed during the current study are available from the corresponding author on reasonable request.
