# Supplementary material for: Targeting myeloperoxidase limits myeloid cell immunosuppression enhancing immune checkpoint therapy for pancreatic cancer
Source: Cancer Immunol Immunother. 2024 Feb 17;73(3):57. doi: 10.1007/s00262-024-03647-z (PMC10874341; doi:10.1007/s00262-024-03647-z)
Supplement: Supplementary file 1 — Supplementary file1 (DOCX 7396 KB) [file 262_2024_3647_MOESM1_ESM.docx]

**Supplementary Materials**

**Targeting myeloperoxidase limits myeloid cell immunosuppression enhancing immune checkpoint therapy for pancreatic cancer**

**Running Title:** Targeting myeloperoxidase enhances immune checkpoint therapy

**Authors:** Angisha Basnet^1^, Kaitlyn M. Landreth^1^, Remi Nohoesu^1^, Stell P. Santiago^3^, Werner J. Geldenhuys^2, 4^, Brian A. Boone^2,5^, Tracy W. Liu^1, 2^ *

**Affiliations:**

^1^ Department of Microbiology, Immunology, and Cell Biology, West Virginia University, Morgantown, WV, 26506, USA.

^2^ WVU Cancer Institute, West Virginia University, Morgantown, WV, 26506, USA.

^3^ Department of Pathology, Anatomy and Laboratory Medicine, West Virginia University, Morgantown, WV, 26506, USA.

^4^ Department of Pharmaceutical Sciences, West Virginia University, Morgantown, WV, 26506, USA.

^5^ Division of Surgical Oncology, Department of Surgery, West Virginia University, Morgantown, WV, 26506, USA.


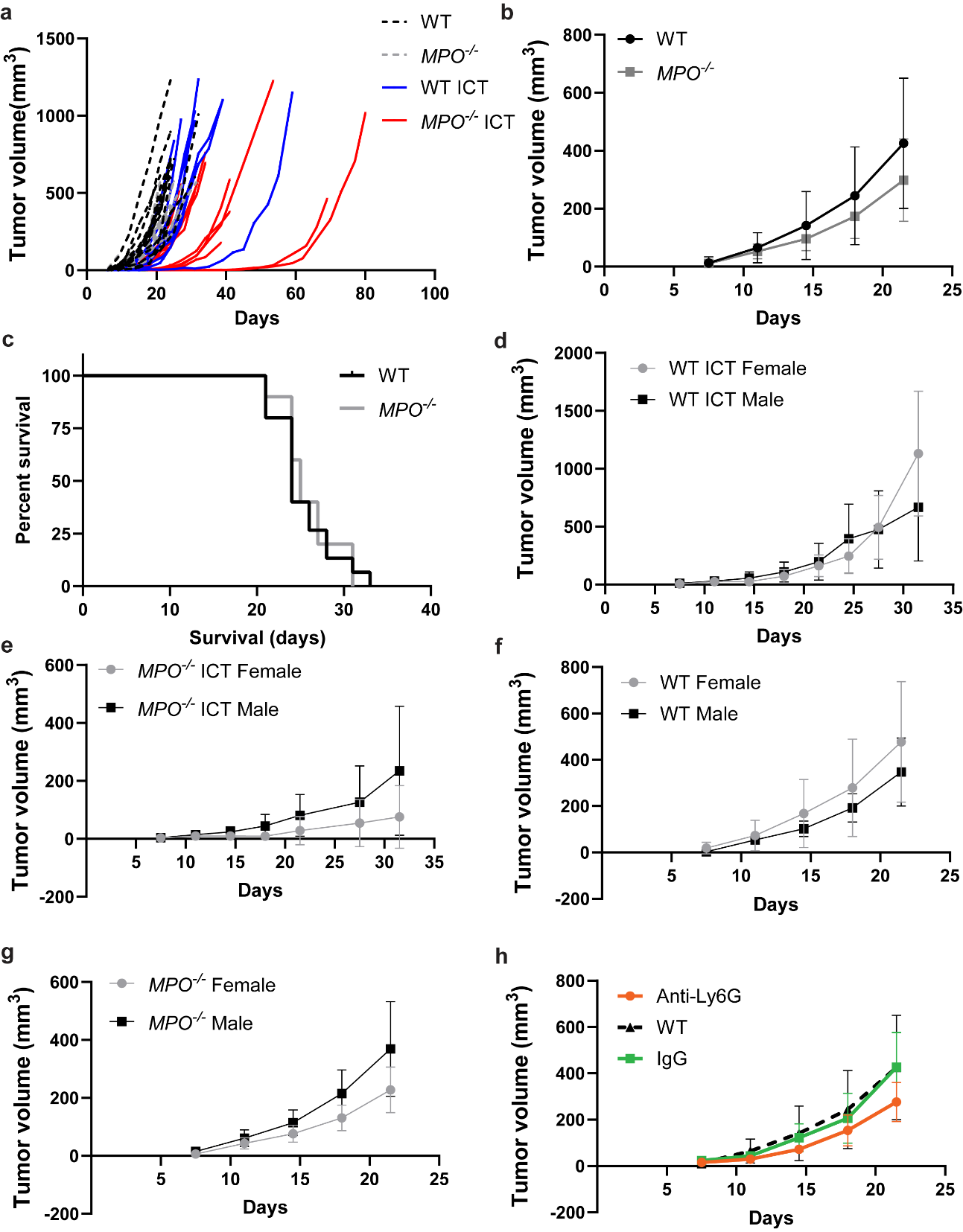


Figure S1. Subcutaneous KPCY6419 tumor growth. (a) Individual tumor growth curves for KPCY6419 tumor-bearing 8-week-old WT and *MPO^-/-^* mice ICT treated and untreated. (b) Tumor volume measurements and (b) survival curves for KPCY6419 tumor-bearing 8-week-old WT and *MPO^-/-^* mice (n = 10 ICT treated mice/group, n = 15 WT mice, n = 10 *MPO^-/-^* mice). Tumor volume measurements comparing male and female (c) ICT treated WT mice (n = 5 male, n = 5 female), (d) ICT treated *MPO^-/-^* mice (n = 5 male, n = 5 female), (e) WT mice (n = 6 male, n = 9 female) and (f) *MPO^-/-^* mice (n = 5 male, n = 5 female). Tumor volume measurements of KPCY6419 tumor-bearing 8-week-old WT mice treated with anti-Ly6G antibody or IgG control (n = 5 mice/group).


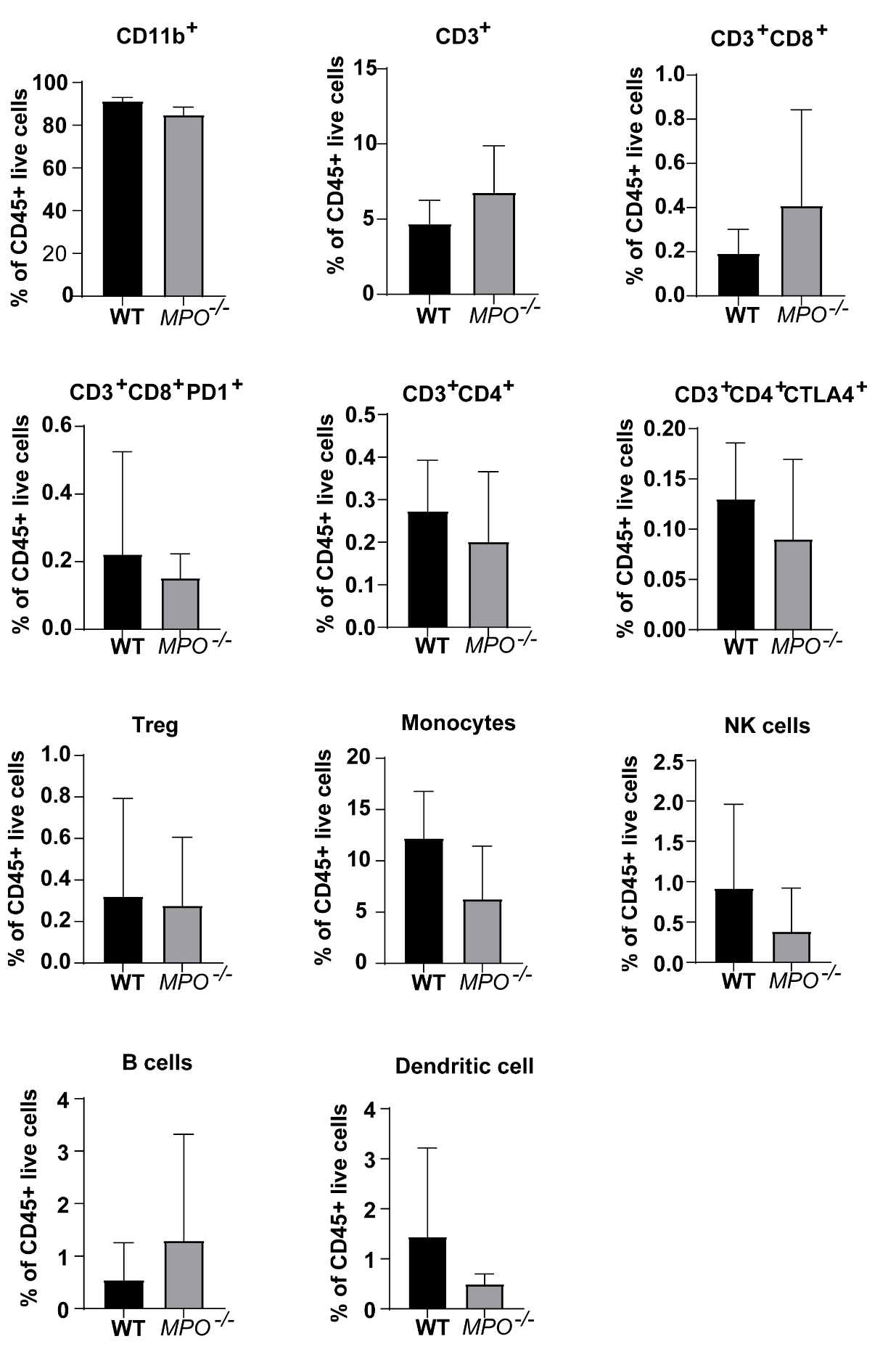


Figure S2. Flow cytometry of endpoint KPCY6419 tumors from WT and *MPO^-/-^* mice (n = 6 mice/group). Data shown as mean ± SD.


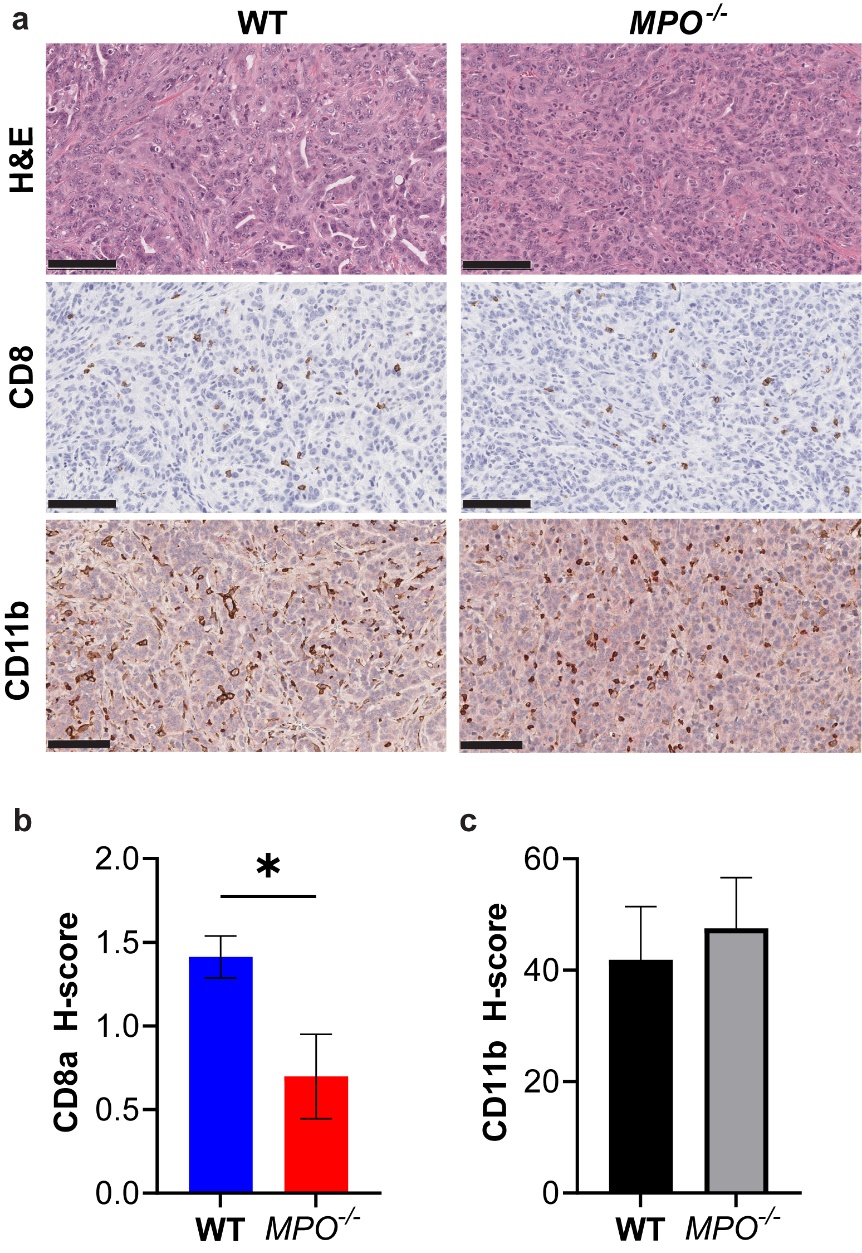


Figure S3. Endpoint histology of KPCY6419 tumors from WT and *MPO^-/-^* mice. Representative (a) histology of H&E, CD8 and CD11b staining and corresponding quantification of (b) CD8a T cells and (c) CD11b cells (n = 3 mice/group).


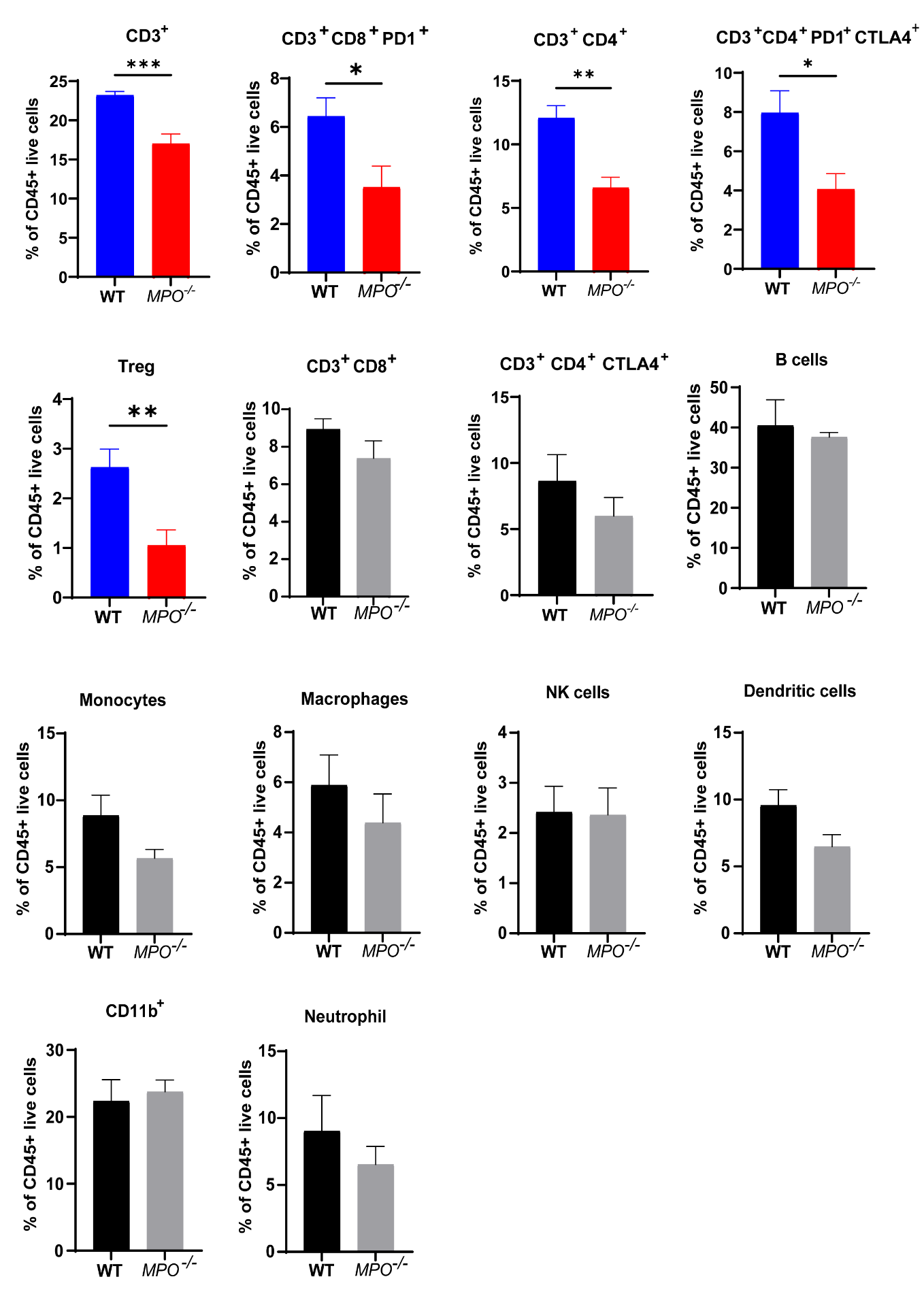


Figure S4. Systemic immune composition. Flow cytometry of spleens at tumor endpoint from WT and *MPO^-/-^* mice (n = 6 mice/group). Data shown as mean ± SD; unpaired student t test, *p<0.05, **p<0.01, ***p<0.001.


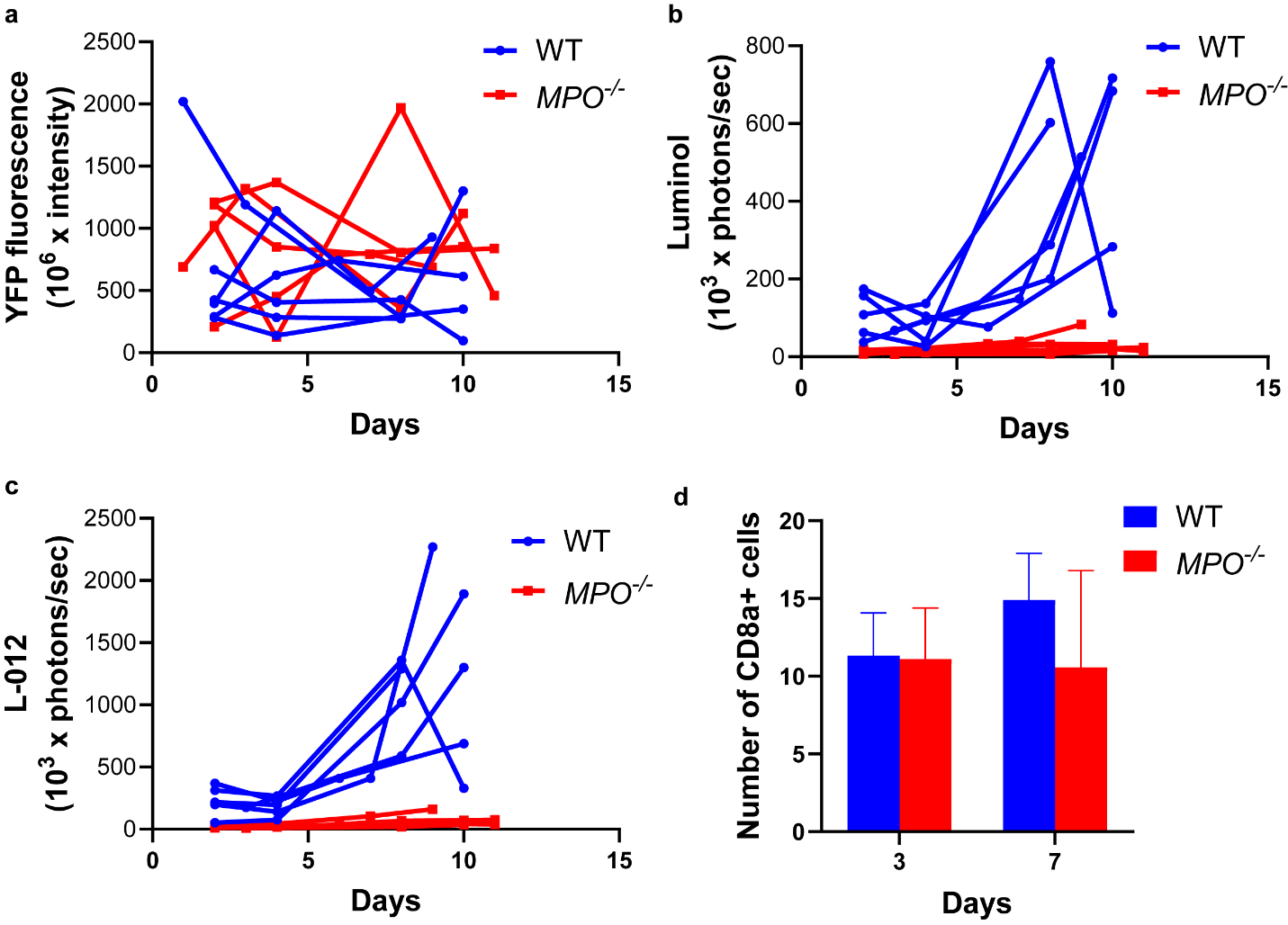


Figure S5. Intravital macro-imaging and microscopic quantification. Longitudinal (a) tumor YFP fluorescence, (b) luminol bioluminescence and (c) L-012 bioluminescence quantification over time (day 2 to day 12) in WT and *MPO^-/-^* mice (n = 6 WT mice, n = 5 *MPO^-/-^* mice). Each line represents a different animal. (d) Quantification of number of CD8^+^ T cells within the tumor (n = 3 images per mouse per time point; n = 24 WT images, n = 30 *MPO^-/-^* images). Data shown as mean ± SD; two-way ANOVA followed by Bonferroni's multiple comparisons test.


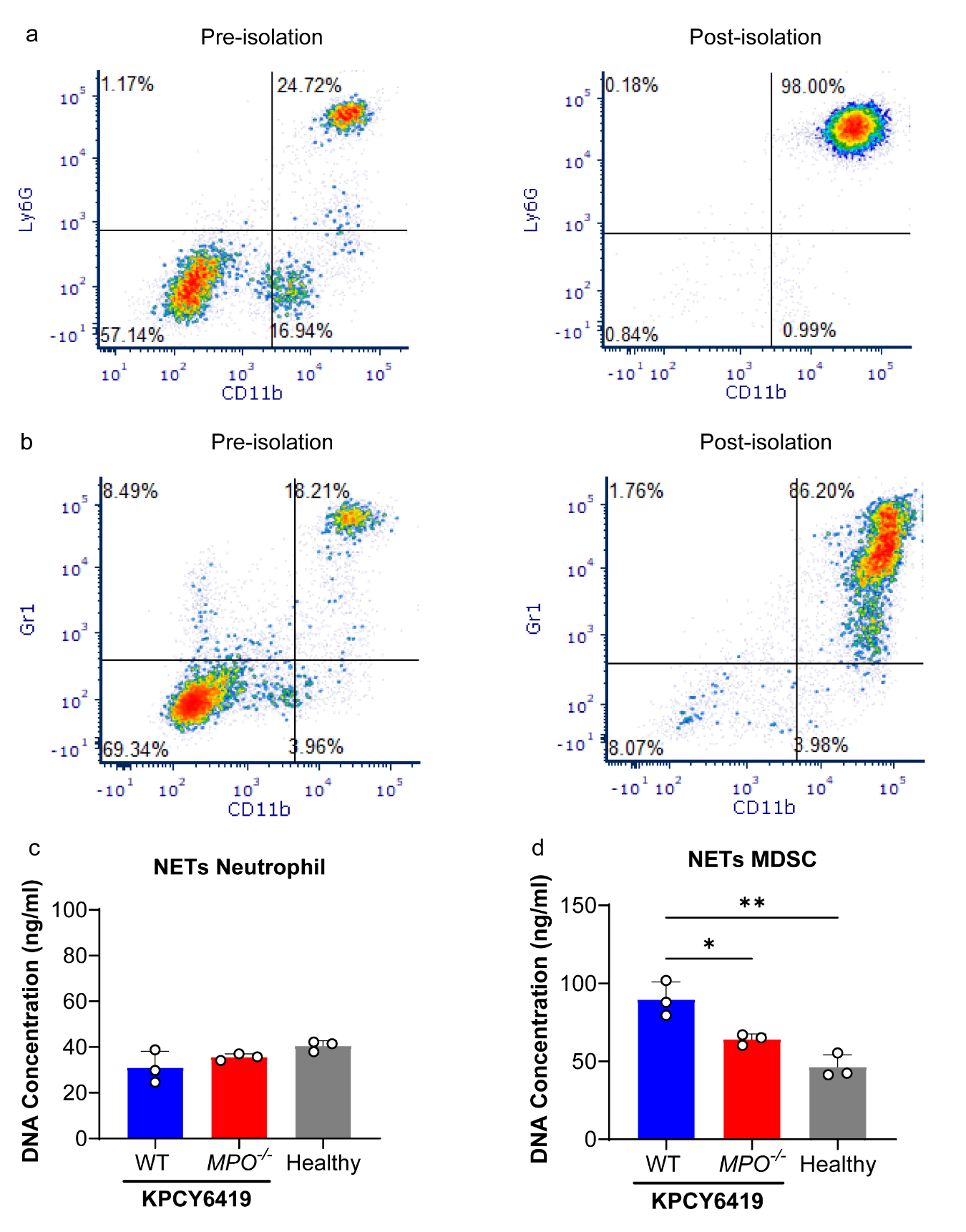


Figure S6. Spleen isolation of neutrophils and MDSCs from PDAC tumor-bearing and healthy mice. Quantification of spleen isolated (a) neutrophil and (b) MDSC purity pre and post isolation kit by flow cytometry. NET quantification in isolated basal (c) neutrophils and (d) MDSCs using Pico Green. Western blot of MPO expression in (e) neutrophils and (f) MDSCs. Data shown as mean ± SD; n = 3 samples per group.


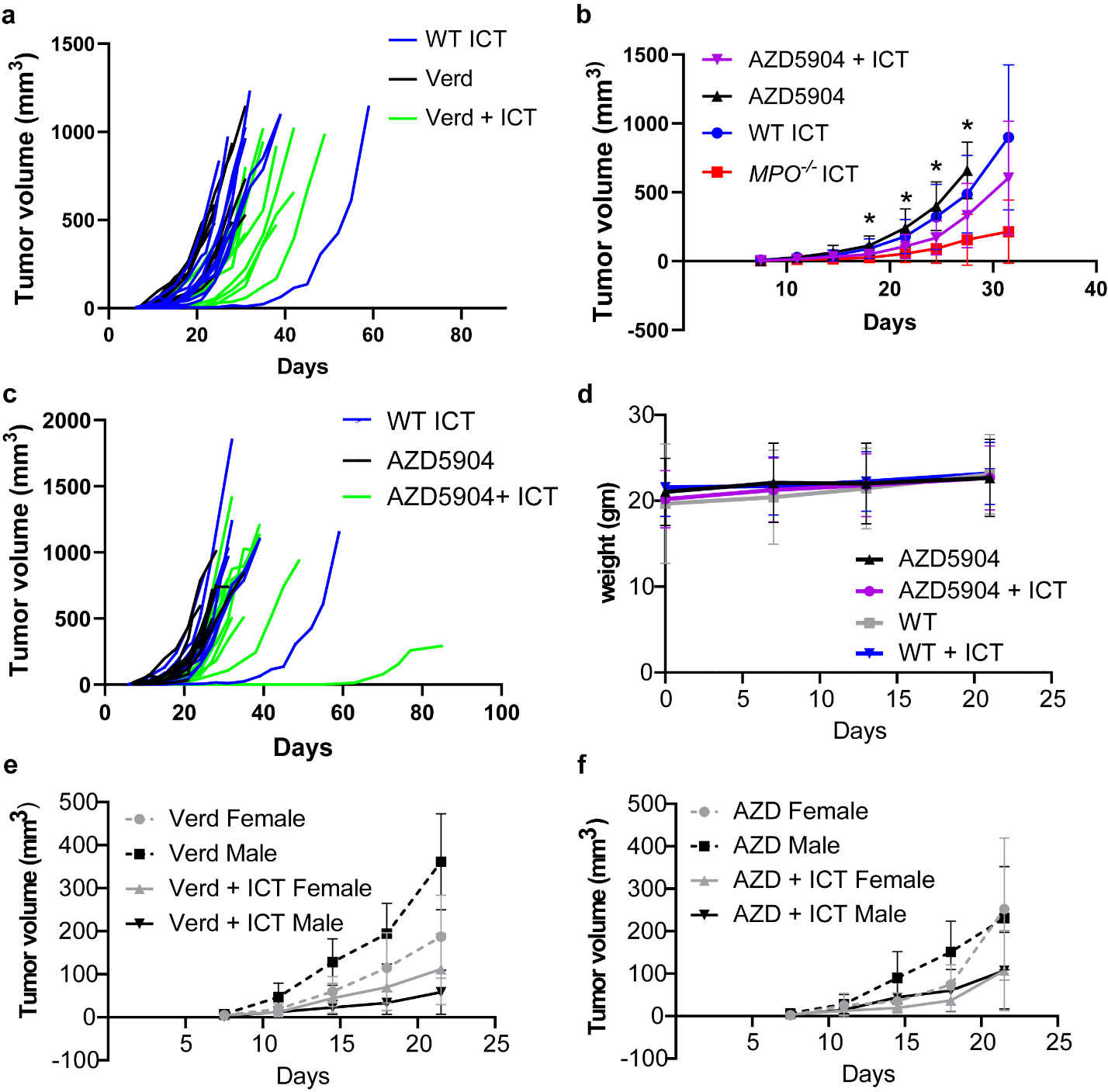


Figure S7. PDAC-bearing WT mice treated with combination ICT and MPO inhibitors. (a) Individual tumor growth curves for verdiperstat (Verd) treated mice (n = 10mice/group). (b) Average tumor volume, (c) individual growth curves and (d) body weight measurements for KPCY6419 tumor-bearing 8-week-old WT treated with AZD5904 with or without ICT (n = 10 mice/group). Data shown as mean ± SD, one-way ANOVA followed by Dunnett’s multiple comparison test, *p<0.05 (black * indicates significant differences between AZD5904 and AZD5904 + ICT). Tumor volume measurements comparing male and female (c) verd and verd + ICT treated WT mice (n = 5 male, n = 5 female) and (d) AZD5904 and AZD5904 + ICT treated WT mice (n = 5 male, n = 5 female). Data shown as mean ± SD.


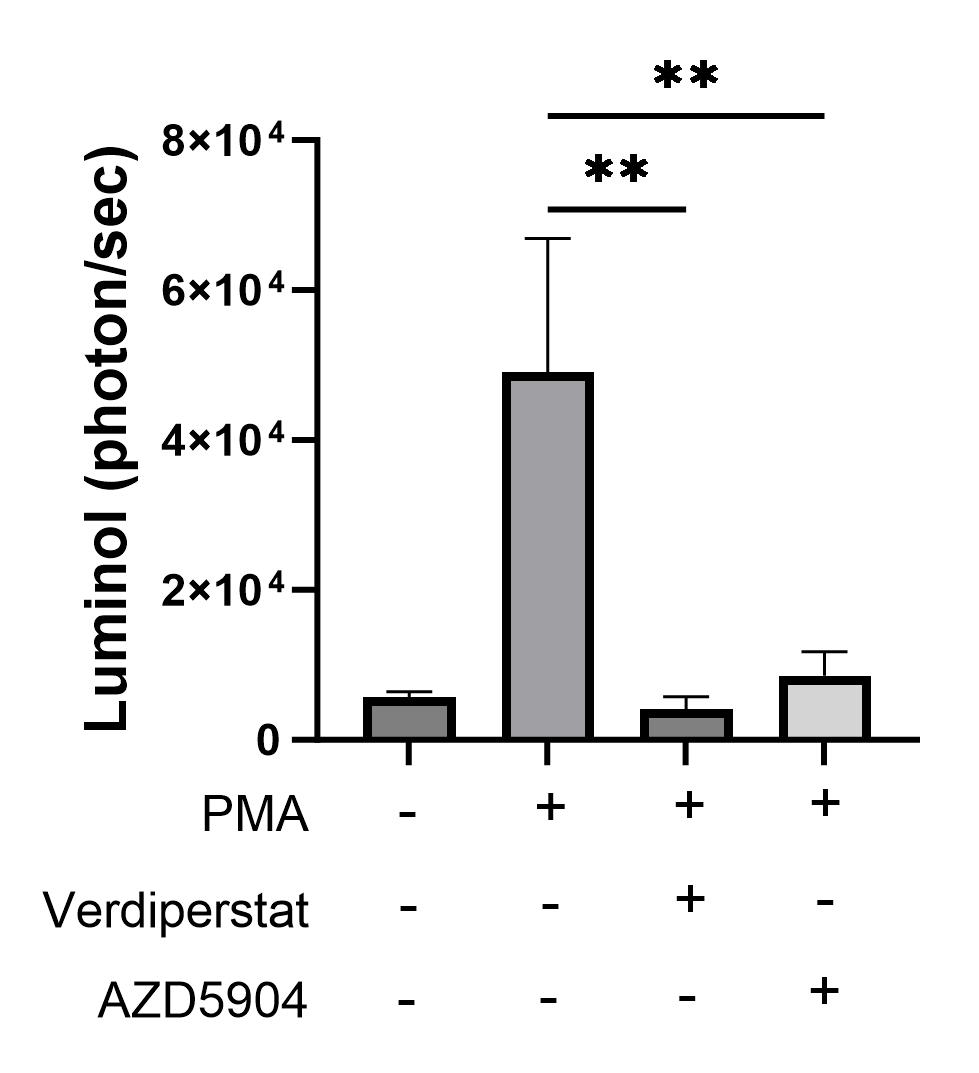


Figure S8. Verdiperstat and AZD5904 significant inhibit MPO activity in PMA stimulated bone marrow isolated immune cells from healthy WT mice. MPO activity was quantified by luminol bioluminescence. Data shown as mean ± SD, one-way ANOVA followed by Dunnett’s multiple comparison test, **p<0.001 (n = 3 samples per group).

a


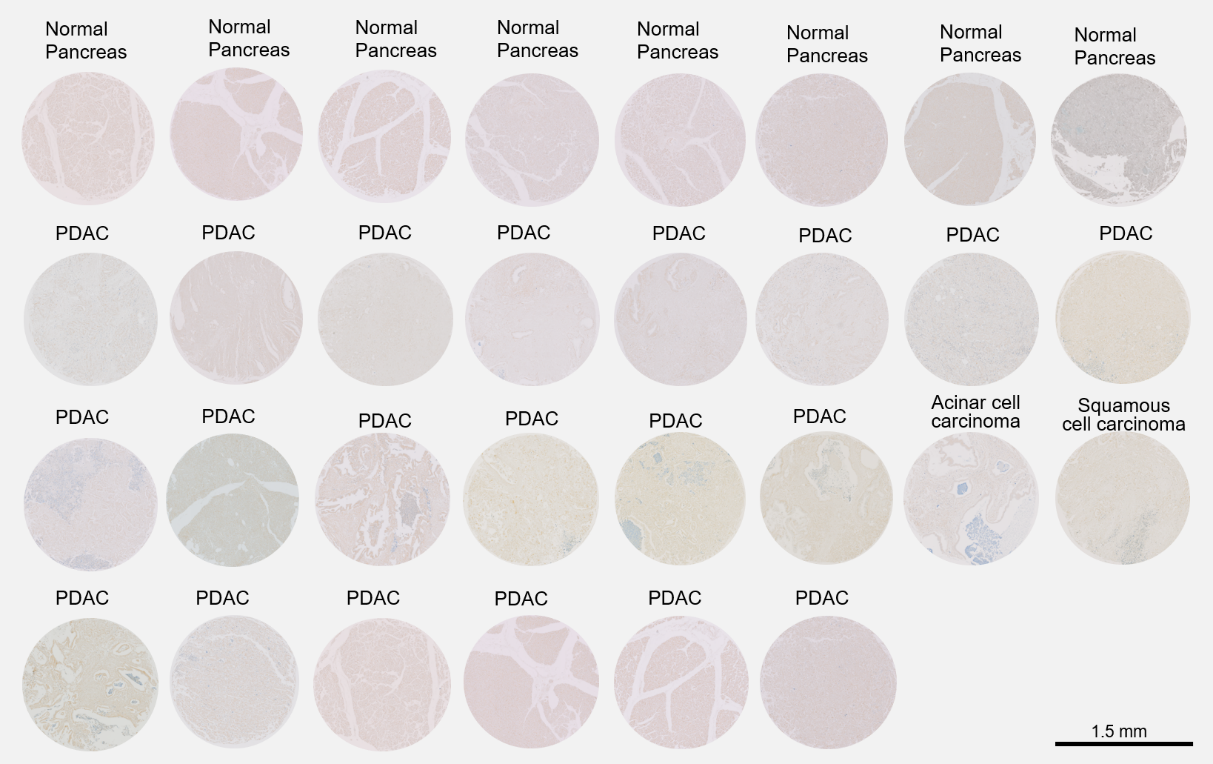


b


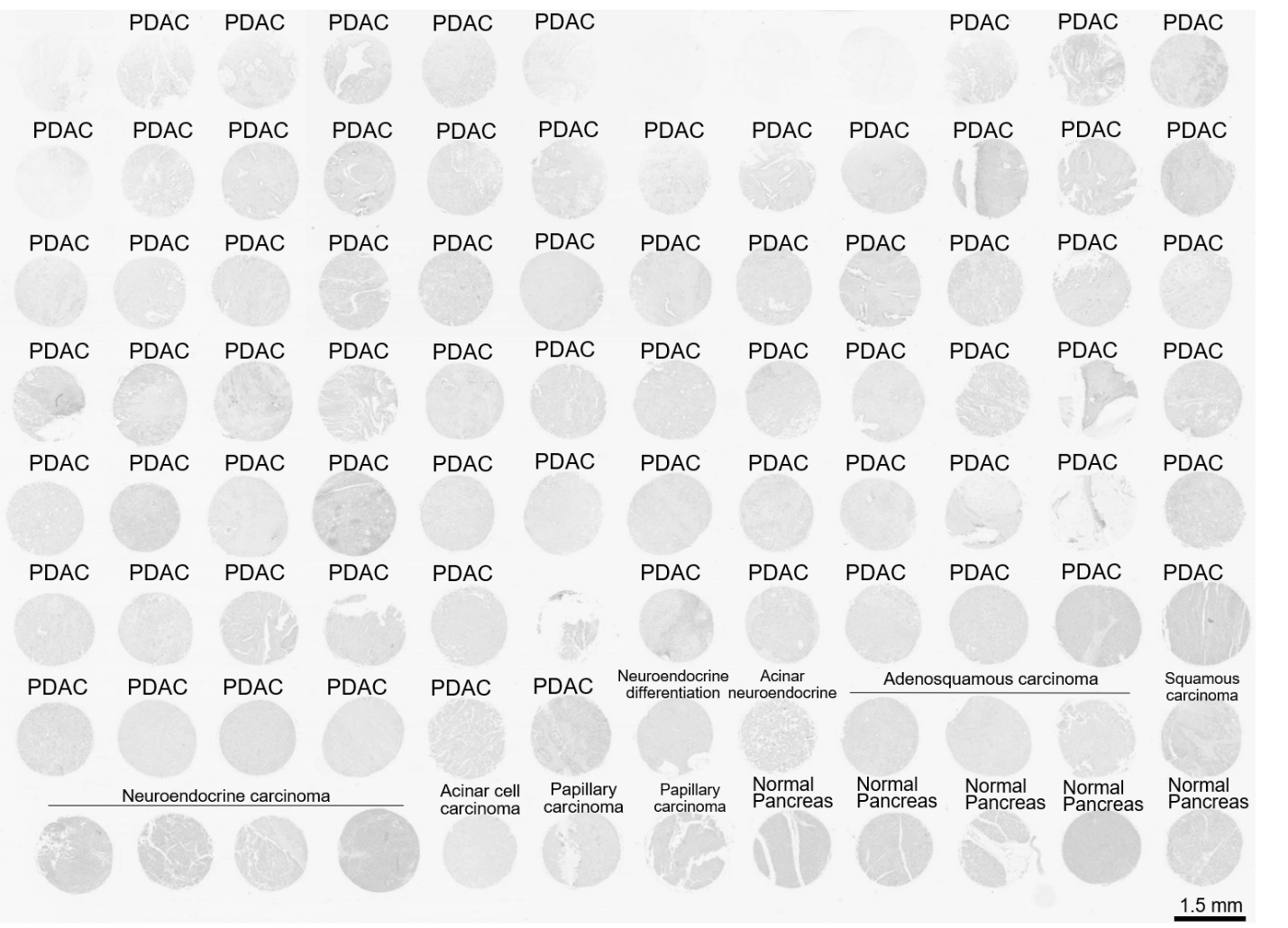


Figure S9. Histological MPO staining of human tissue microarray of (a) PA483e and (b) PA961f.


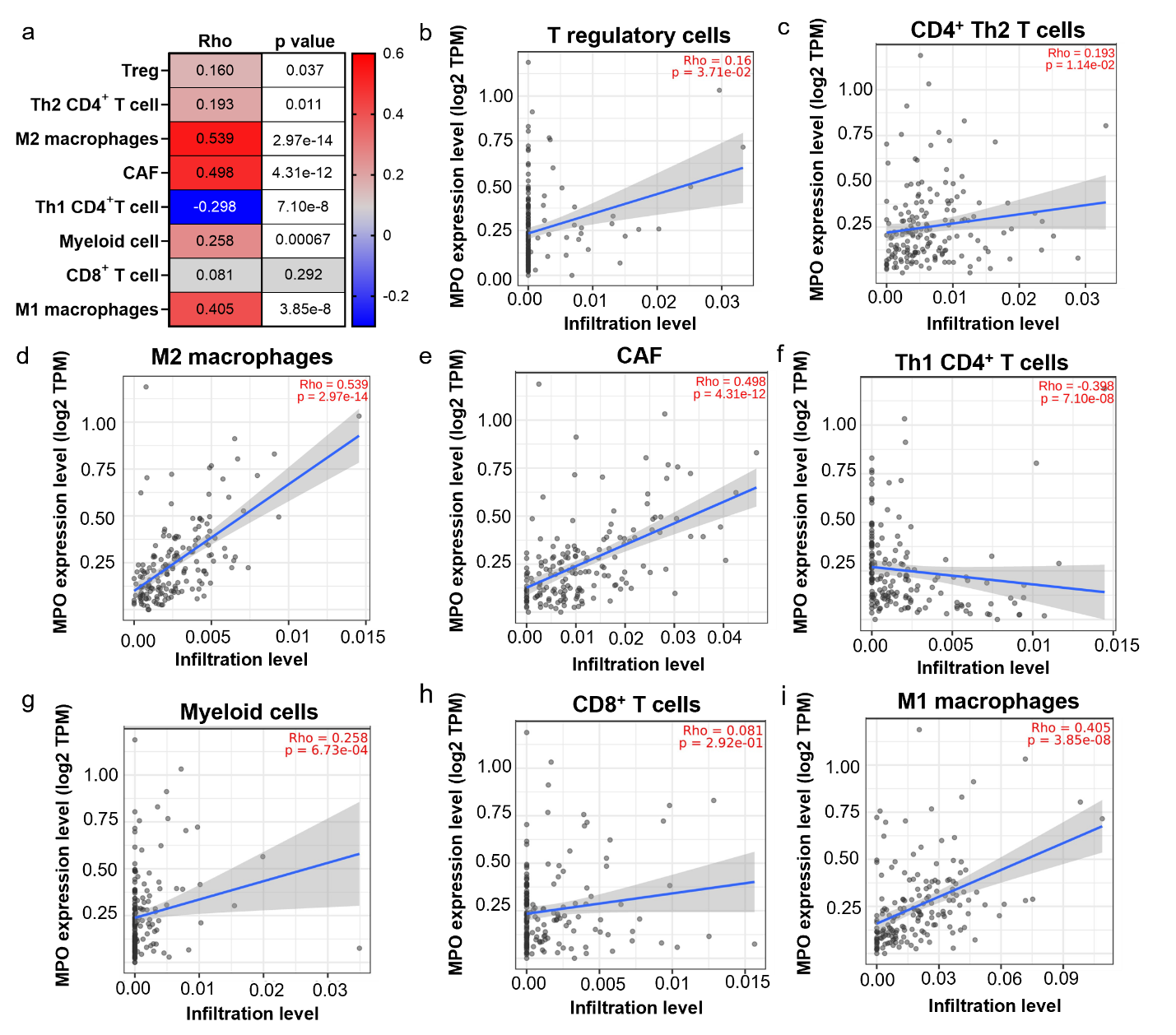


Figure S10. MPO gene expression correlates with immunosuppressive immune cell subsets using the TCGA pancreatic adenocarcinoma (PAAD) data set. (a) Heatmap of the correlation (rho) and p values of MPO gene expression with various estimated immune infiltration levels (red is positively correlated, blue is negatively correlated and grey are insignificant with p>0.05 values). Corresponding correlation graphs of (b) T regulatory cells (Treg), (c) Th2 CD4^+^ T cell, (d) M2 macrophage, (e) cancer associated fibroblast (CAF), (f) Th1 CD4^+^ T cell, (g) myeloid cells, (h) CD8^+^ T cell, and (i) M1 macrophages. Values and graphs were generated using the TIMER2.0 XCell algorithm.


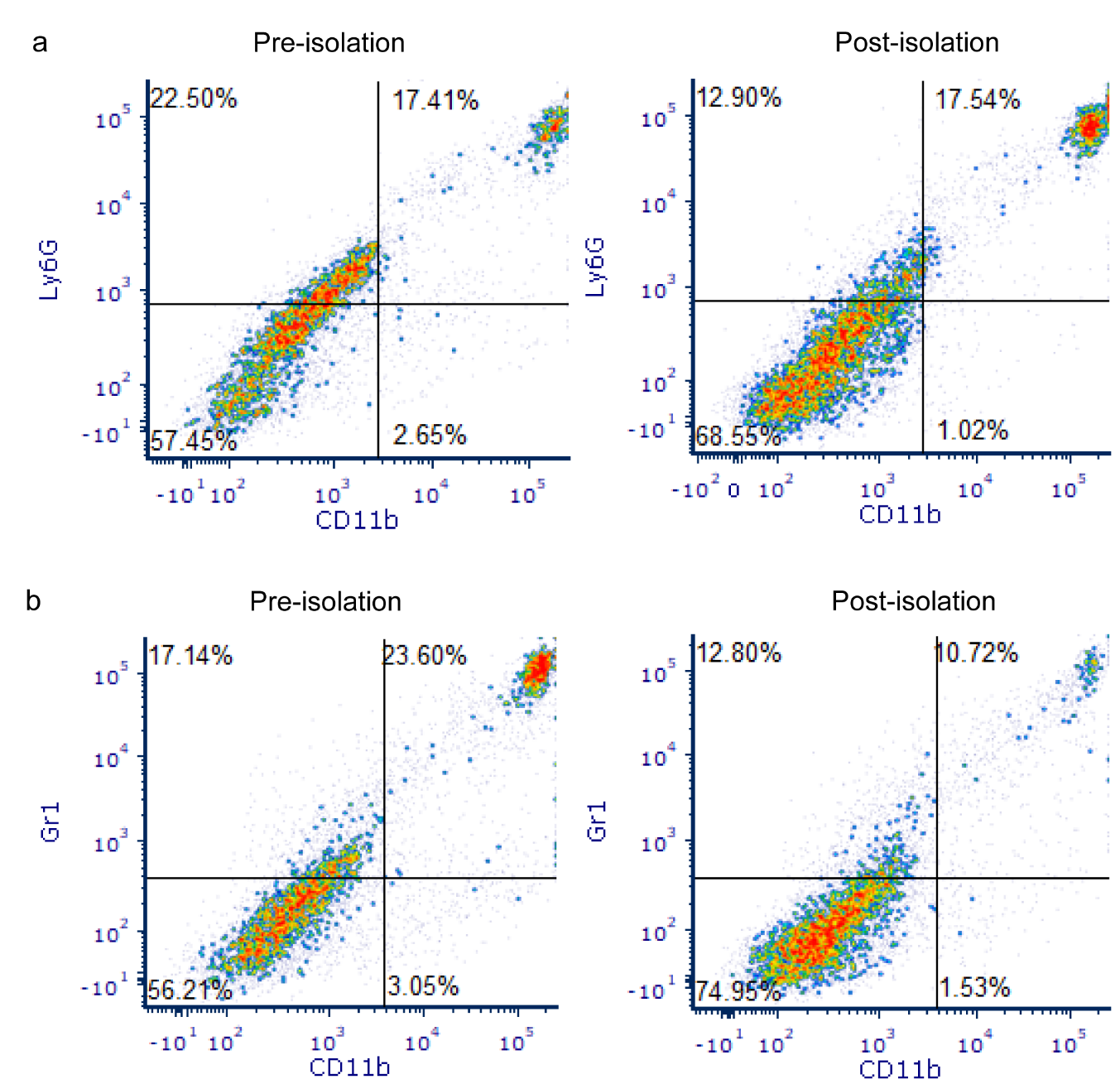


Figure S11. Minimal purification of isolated neutrophils and MDSCs from subcutaneous KPCY6419 tumors. Representative purity of tumor isolated (a) neutrophil and (b) MDSC pre and post isolation kit by flow cytometry.


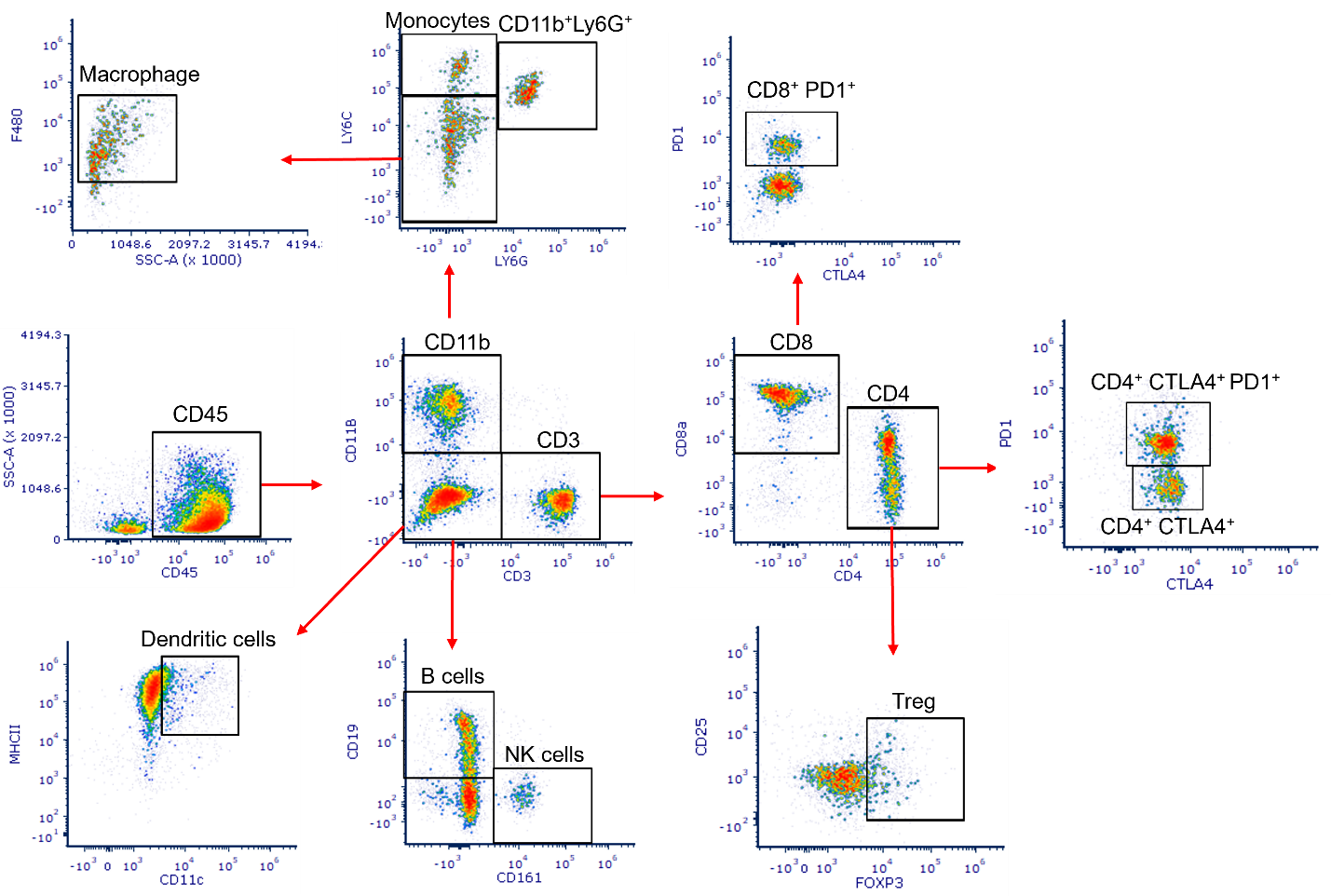


Figure S12. Representative flow cytometry gating scheme identifying the various immune cell subsets.

| **Antibody** | **Fluorophore** | **Company** |
| --- | --- | --- |
| CD11b | Spark NIR 685 | BioLegend (CA, USA) |
| CD45r | Spark Blue 550 | BioLegend (CA, USA) |
| MHC II | PE/Fire 640 | BioLegend (CA, USA) |
| CD161 (NK) | BV 711 | BioLegend (CA, USA) |
| CD11c | BV 421 | BioLegend (CA, USA) |
| F480 | APC/Fire 810 | BioLegend (CA, USA) |
| PDL1 | APC | BioLegend (CA, USA) |
| Ly6G | PerCP/Cy 5.5 | BioLegend (CA, USA) |
| Ly6C | BV 785 | BioLegend (CA, USA) |
| PD-1 | PE/Dazzle | BioLegend (CA, USA) |
| CD115 | Pe Cy7 | BioLegend (CA, USA) |
| CD45 | Pac Blue | BioLegend (CA, USA) |
| L/D | Zombie Aqua | BioLegend (CA, USA) |
| CTLA4 (CD152) | BV 421 | BioLegend (CA, USA) |
| FOXP3 | PE | BioLegend (CA, USA) |
| CD49b | PerCP/CY 5.5 | BioLegend (CA, USA) |
| CD25 | PE CY7 | BioLegend (CA, USA) |
| CD45r | Spark Blue 550 | BioLegend (CA, USA) |
| CD19 | APC/Fire 810 | BioLegend (CA, USA) |
| CD4 | BV 785 | BioLegend (CA, USA) |
| CD8a | APC/Fire 750 | BioLegend (CA, USA) |
| CD3 | Alexa Fluor 488 | BioLegend (CA, USA) |

Table S1. List of antibodies used for flow cytometry studies.
